# Study protocol: childhood outcomes of fetal genomic variants: the PrenatAL Microarray (PALM) cohort study

**DOI:** 10.1186/s12887-021-02809-7

**Published:** 2021-10-11

**Authors:** Lisa Hui, Cecilia Pynaker, Joanne Kennedy, Sharon Lewis, David J. Amor, Susan P. Walker, Jane Halliday, On behalf of the PALM cohort study group, Fiona Norris, Lucy Gugasyan, Matt Regan, Anand Vasudevan, Susan Fawcett, George McGillivray, Melissa Graetz, Joanne Said, Lisa Begg, Ron Wapner, Brynn Levy

**Affiliations:** 1 Reproductive Epidemiology group, Murdoch Children’s Research Institute, Flemington Rd, Parkville, VIC Australia; 2 Department of Obstetrics and Gynaecology, University of Melbourne, Parkville, VIC Australia; 3 Mercy Hospital for Women, Heidelberg, VIC Australia; 4 Northern Health, Epping, VIC Australia; 5 Department of Paediatrics, University of Melbourne, Parkville, VIC Australia; 6 Neurodisability and Rehabilitation group, Murdoch Children’s Research Institute, Flemington Rd, Parkville, VIC Australia

**Keywords:** Prenatal diagnosis, Chromosomal microarray analysis, Genomic copy number variant, Variants of uncertain significance, Childhood outcomes, Postnatal outcomes

## Abstract

**Abstract:**

**Background:**

The implementation of genomic testing in pregnancy means that couples have access to more information about their child’s genetic make-up before birth than ever before. One of the resulting challenges is the management of genetic variations with unclear clinical significance. This population-based study will help to close this critical knowledge gap through a multidisciplinary cohort study of children with and without genomic copy number variants (CNVs) diagnosed before birth. By comparing children with prenatally-ascertained CNVs to children without a CNV, we aim to (1) examine their developmental, social-emotional and health status; (2) measure the impact of prenatal diagnosis of a CNV on maternal perceptions of child health, behavior and development; and (3) determine the proportion of prenatally-ascertained CNVs of unknown or uncertain significance that are reclassified as benign or pathogenic after 2 or more years.

**Methods:**

This study will establish and follow up a cohort of mother-child pairs who have had a prenatal diagnosis with a chromosomal microarray from 2013-2019 in the Australian state of Victoria. Children aged 12 months to 7 years will be assessed using validated, age-appropriate measures. The primary outcome measures will be the Wechsler Preschool and Primary Scale of Intelligence IV (WPSSI-IV) IQ score (2.5 to 7 year old’s), the Ages and Stages Questionnaire (12-30 months old), and the Brief Infant- Toddler Social and Emotional Assessment (BITSEA) score. Clinical assessment by a pediatrician will also be performed. Secondary outcomes will be scores obtained from the: Vineland Adaptive Behavior Scale, Maternal Postnatal Attachment Questionnaire, the Vulnerable Child Scale, Profile of Mood States, Parent Sense of Competence Scale. A descriptive analysis of the reclassification rates of CNVs after ≥2 years will be performed.

**Discussion:**

This study protocol describes the first Australian cohort study following children after prenatal diagnostic testing with chromosomal microarray. It will provide long-term outcomes of fetal genomic variants to guide evidence-based pre-and postnatal care. This, in turn, will inform future efforts to mitigate the negative consequences of conveying genomic uncertainty during pregnancy.

**Trial registration:**

ACTRN12620000446965p; Registered on April 6, 2020.

**Supplementary Information:**

The online version contains supplementary material available at 10.1186/s12887-021-02809-7.

## Background

Congenital anomalies, of which one third are chromosomal in origin, are the leading cause of perinatal death in Australia [1]. Prenatal screening for fetal chromosomal conditions is thus an important component of care for the 300,000 women who give birth in Australia each year. National guidelines recommend that voluntary screening, and the option of follow-up prenatal diagnosis, be provided to all pregnant women [2]. In 2019, Victoria recorded 409 prenatal diagnoses of major chromosome conditions, in a state population with 76,000 births per year (1 in 186 births) [3].

Over the past decade, technological advances have revolutionized genetic testing at every stage of reproduction [4]. Classical cytogenetics has been superseded by chromosomal microarray (CMA) as the gold standard for chromosomal assessment [5]. CMA interrogates the genome at a higher resolution than conventional karyotyping, allowing previously undetected submicroscopic copy number variants (CNVs) to be detected. A National Institute of Child Health and Human Development (NICHD) multicenter trial in the United States (US) provided the first large-scale report on the use of CMA for fetal chromosome analysis [6]. This landmark trial compared the accuracy of prenatal CMA to that of standard karyotyping and found that 5.8% of pregnancies with a fetal structural anomaly and a normal karyotype had a clinically significant CNV detected. Professional societies now recommend the use of prenatal CMA, particularly for the investigation of fetal structural anomalies [2, 7]. This has rapidly transitioned to being the ‘default’ investigation for any prenatal diagnostic test and accordingly in Victoria, the annual proportion of prenatal diagnoses utilizing CMA has exceeded 80% since 2015 [3].

The genomic imbalances detected by CMA include pathogenic CNVs (pCNVs) that are associated with well-described syndromes such as the 22q11.2 deletion syndrome and Prader-Willi / Angelman syndromes. However, with the ability of CMA to detect smaller genomic imbalances than conventional karyotype, comes a correspondingly higher detection rate of uncertain findings [8]. These variants of uncertain (or unknown) significance (VUS) are CNVs that have a variable amount of evidence available to assess their pathogenicity. Parental testing is often required to assist in interpretation of fetal VUS.

Fetal VUS present special clinical and ethical challenges compared with the pediatric setting [9]. VUS are reported in 5% of prenatal samples in Victoria, resulting in almost 100 women each year being informed that their unborn baby has an uncertain diagnosis [10]. These VUS include CNVs that may predispose to developmental disability or neuropsychiatric illness, but which are highly variable and unpredictable in effect (“neurosusceptibility CNVs”) [11]. Interpretation of uncertain variants during pregnancy requires more caution because where there is no apparent fetal phenotype, this information may bring more harms than benefits. There is currently no guidance for structured assessment and follow-up for these children after birth. Furthermore, our understanding of the clinical significance of variants is continuously evolving, and classification of CNVs may change over time without patients being notified of updated information.

Long-term outcomes of prenatally-ascertained CNVs are needed. To our knowledge, worldwide, there are only two cohort studies in progress investigating the postnatal outcomes of prenatal CNVs detected on CMA – the NICHD sponsored cohort from the United States [6] and the Belgium MicroArray Prenatal (BEMAPRE) consortium [12]. Generating new knowledge on long-term outcomes of fetal genomic variants will have a positive health impact by supporting (i) evidence-based pre- and postnatal care, and (ii) mitigating the negative consequences of conveying genomic uncertainty during pregnancy.

## Methods/Design

### Aims

The PALM cohort study will establish and follow-up a cohort of mother-child pairs who had a prenatal diagnosis of a CNV from 2013-2019 (inclusive) in the Australian state of Victoria. Victoria has approximately 78,000 births per year and a median maternal age of 31.5 years [13]. Approximately 2-3% of all pregnant women undergo prenatal diagnosis per year [14].

The study aims are to:

1. Compare the developmental, social-emotional and health status of children with a prenatal diagnosis of a prenatal CNV with those children with no CNV detected on CMA.
2. Measure the impact of a prenatal diagnosis of a CNV on maternal perceptions of child health, behavior, and development.
3. Determine the proportion of CNV ascertained prenatally that are reclassified from unknown or uncertain to benign or pathogenic after two or more years using contemporaneous curation resources.

### Study design

This cohort study that will recruit participants with an existing record of a prenatal CMA in the Victorian Prenatal Diagnosis Database (VPDD) between January 2013 and December 2019 inclusive. The VPDD is a unique population-based dataset housed at the Murdoch Children’s Research Institute (MCRI) that has been described in detail in prior publications [15]. The women who underwent prenatal diagnosis during the study period, and who had a live child at the time of discharge from hospital after birth, will be approached by mail from their clinical referrer to offer participation in the study. In brief, study participation will involve providing permission for data collection from the health records, completion of questionnaires, and an assessment of their child’s health and development.

### Study population

Each year the VPDD receives prenatal diagnostic testing results on approximately 150 pregnancies with CNVs, and 1000 pregnancies with no CNVs. We will recruit an equal number of participants with and without CNVs, frequency matched for fetal sex, trimester at prenatal diagnosis, maternal age group (by decade) and calendar year of testing. Due to the modifier of structural anomaly on childhood outcomes, women with fetal structural abnormalities will be excluded from the control group of pregnancies with no CNVs.

### Inclusion criteria

The biological mother (gestational carrier) is the most relevant parent/legal guardian to consent for participation as she was the individual that underwent the prenatal diagnostic procedure. She will be eligible by meeting the following criteria:

- Prenatal CMA result reported in a singleton pregnancy during January 2013 - December 2019 inclusive
- At least 18 years of age at enrolment
- Able to provide informed consent in English
- Live infant outcome from index pregnancy
- Primary care-giver for the child at the time of hospital discharge after birth
- Capable of providing consent on behalf of herself and her child
- Able to complete the parental questionnaires and accompany her child for study assessments
- Resides in Victoria

### Sample size

The VPDD from 2013-2019 has recorded an average of 147 CNVs (85 VUS, 62 pCNVs) each year. Our total potential CNV cohort for the proposed 7-year study period will be approximately 1029.

We expect losses of potential recruits due to failed contact tracing, language ineligibility, perinatal loss, and refusal of consent, and therefore conservatively estimate a final sample size of 354 women for the CNV cohort. This is based on 45% recruitment of women with fetal VUS (n=267), and 20% recruitment rate for pathogenic CNVs (n=87). This is in line with participation rates in the NICHD microarray cohort where they achieved 41%, 56% and 70% participation rates for those with pathogenic, VUS or benign/normal CMA results respectively [16]. There are 7155 potential eligible controls in the study period. We will require 5% of women with normal CMA results to be recruited to form the control cohort (n=358).

### Recruitment of potential participants

**Fig. 1:**
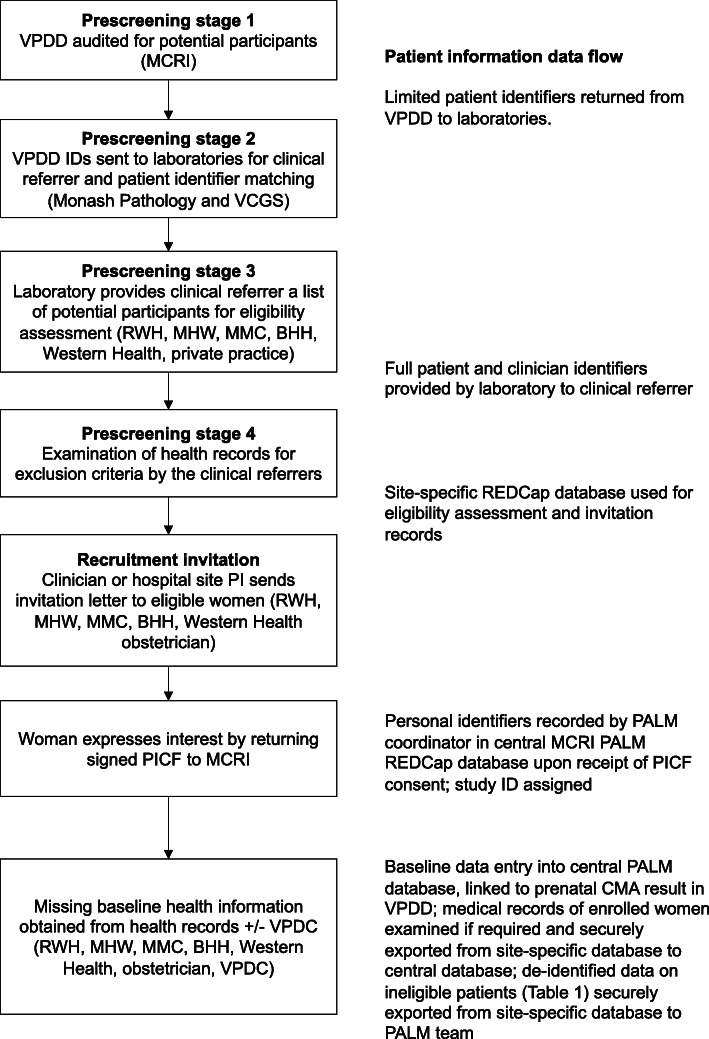
Recruitment strategy. Abbreviations: BHH (Box Hill Hospital); CMA (chromosomal microarray analysis); ID (Identifier); MCRI (Murdoch Children’s Research Institute); MHW (Mercy Hospital for Women); MMC (Monash Medical Centre); PALM (PrenatAL Microarray); PI (Principle Investigator); PICF (Participant Information Consent Form); RWH (Royal Women’s Hospital); VPDC (Victorian Perinatal Data Collection); VPDD (Victorian Prenatal Diagnosis Data collection)

This will involve a multi-step pre-screening stage to maintain patient privacy and minimize the chance of approaching ineligible women to participate (Fig. 1).

**Fig. 2:**
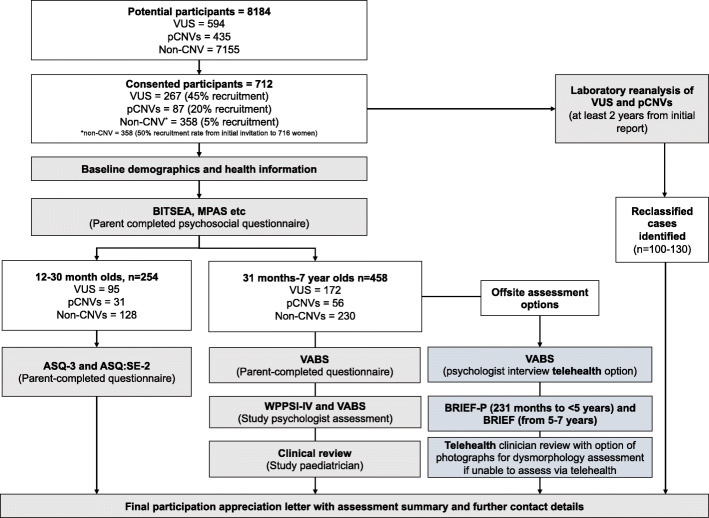
Participant flowchart including estimated recruitment rates by CNV subgroup (pathogenic CNV or variant of uncertain significance). Abbreviations: ASQ:SE-2 (Ages and Stages Questionnaire: Social-Emotional-2); ASQ-3 (Ages and Stages Questionnaire, third edition); BITSEA (Brief Infant-Toddler Social and Emotional Assessment); BRIEF (Behaviour Rating Inventory of Executive Function); BRIEF-P (Behaviour Rating Inventory of Executive Function – Preschool version); pCNVs (pathogenic copy number variant); MPAS (Maternal Postnatal Attachment Questionnaire); VABS (Vineland-II Adaptive Behaviour Scale); VUS (variants uncertain/unknown significance); WPPSI-IV (Wechsler Preschool and Primary Scale of Intelligence IV)

### Pre-screening

The aim of the pre-screening stage is to accurately identify potential eligible participants, and to avoid contacting women who may have experienced a perinatal loss in the relevant pregnancy. For this reason, the involvement of the clinical referrer in the recruitment process is considered essential.

Pre-screening will include the following:

(i) Pre-screening stage 1. Audit of prenatal CMA results at the VPDD to identify potential participants based on prenatal diagnosis results. This will be performed by the study team at the MCRI who manage the VPDD. A list containing all records with a CNV detected on CMA during the study period will be generated and categorized into pCNV or VUS groups. A list of non-CNV records, where there is no fetal structural abnormality, will also be created as the control cohort. Each record will have the limited individual identifiers from the contributing cytogenetic laboratories, available under the existing VPDD ethics protocol (i.e., first three letters of maternal surname and first name, date of testing, type of test, date of birth).

(ii) Pre-screening stage 2. This list of potential participants from the VPDD with the limited identifiers will be sent in a secure file to the respective laboratories that performed the CMA testing using Research Electronic Data Capture (REDCap), a secure web application specifically designed to support online and offline data capture for research studies [17, 18].

(iii) Pre-screening Stage 3. Individual lists of potential participants for each clinical practice location will be generated by the participating laboratories. The vast majority will be captured through the five public hospital fetal medicine units: The Royal Women’s Hospital, The Mercy Hospital for Women, Monash Medical Centre, Sunshine Hospital and Box Hill Hospital. Approximately 20% will be from private obstetricians and regional referrals.

(iv) Pre-screening Stage 4. Examination of health records for exclusion criteria by the clinical referrers. The protocol will vary depending on whether the referral came from a public hospital or private practice.

#### Public hospital fetal medicine units

The lead prenatal genetic clinician and/or the senior genetic counsellor at each of the five fetal medicine units will act as site Principal Investigator (PI) (see acknowledgements for full list of site PIs and associate investigators) and oversee any data collection. The medical records of potential participants sent as a listing from the laboratory will be examined by the site PI to identify those that are eligible. They will also collect deidentified data on ineligible individuals to determine the attrition rate and frequency of factors that lead to ineligibility (Table 1).

**Table 1:** Data elements collected on ineligible individuals

Pregnancy results in perinatal loss (miscarriage, stillbirth, neonatal death, or termination of pregnancy)	
Interpreter required (non-English speaking); language	
Woman unable to given informed consent for medical care	
Year of prenatal diagnosis	
Multiple pregnancy	
Relocation interstate during pregnancy	
Mother not intended to be primary care-giver of child	
Unable to be assessed (missing data)	

For those women that did not give birth in the fetal medicine unit where the prenatal diagnosis took place (e.g., women from regional Victoria referred to tertiary units for prenatal diagnosis), the planned hospital of birth and the clinical referrer will be recorded by the PI. A letter or email from study team will be sent to the original clinical referrer to ask if their participant meets the eligibility requirements for the study.

#### Private practice referrers

The laboratory will send either a letter or email regarding potentially eligible recruit(s) to the private obstetrician who referred their patient(s) for prenatal diagnosis. The private practice clinician will be asked to perform an assessment of their patient’s eligibility.

### Recruitment and enrolment

All women who meet the eligibility criteria will be sent an invitation letter and a Participant Information & Consent Form (PICF) by post +/- email (if available) from the clinical referral source. All eligible women in the CNV group will be invited. Based on the above estimated participation rates, we will initially invite 716 women in the non-CNV group, with further invitations to be sent if we do not achieve our target sample size in the control group (Fig. 2). Those who wish to participate will be asked to complete an online consent form in REDCap, accessed via a QR code in the invitation letter. Alternatively, a hard copy consent from can be completed and sent to the Murdoch Children’s Research Institute (MCRI) either via email or in an enclosed postage-paid envelope. The MCRI study coordinator will be the primary contact for all women who are considering participation.

A single reminder letter will be sent three weeks after the initial invitation. If there is no response after this, no further contact will be attempted.

### Ineligible and non-responding individuals

De-identified data will be maintained on all individuals who are deemed ineligible during the pre-screening stage in order to ascertain the representativeness of our cohort and its generalizability to our prenatal diagnosis population. Those women who were invited but did not provide consent to the MCRI team will be considered “non-responders”. We will not collect individual identifiers of these women.

### Screening and baseline health assessment

Baseline variables will be collected directly from the woman by telephone, online survey, or hardcopy postage according to the participant’s preference.

These will include:

- A review of her history to confirm eligibility and willingness to perform the study procedures (parent-completed questionnaires +/- child study visits)
- Baseline demographic and health assessment data
  - Maternal and household information
  - Pregnancy and newborn outcomes
  - Maternal state and trait anxiety
  - Maternal health literacy
  - Health care utilization
- Missing baseline pregnancy outcomes may be collected from health service medical records and/or via linkage to the Victorian Perinatal Data Collection. Women will be asked to provide specific optional consent for data linkage to the Victorian Perinatal Data Collection (VPDC) [19].
- Women will be asked to provide specific optional consent for access to the School Entrant Health Questionnaire (SEHQ) at the time of consenting to participate in the PALM study. This is a parent report instrument that records parent’s concerns and observations about their child’s health and wellbeing as the child begins primary school in Victoria. A separate application to the Department of Education will be made for access to these data on those participants providing specific consent.

### Data collection

Child outcome data will be collected via parent-completed quantitative surveys using validated measures and through psychologist and/or pediatrician assessment. See Table 2 for an overview of study instruments used for each outcome measure. The study data will be collected in 5 main forms.

1. Parent-completed questionnaires – off-site, in parent’s own time
2. Vineland Adaptive Behavior scale (VABS) – telephone interview with mother conducted by a study psychologist
3. WPPSI-IV – child assessment conducted on-site by a study psychologist at the Melbourne Children’s Trials Centre, Parkville, Melbourne
4. Clinical assessment by study pediatrician – conducted on-site at the same visit as the WPPSI-IV wherever practicable.
5. Laboratory reanalysis of the prenatal CNV result - no interview or participant attendance required.

**Table 2:** Overview of study instruments used in the PALM study for children aged 12 months to 7 years

Data collection method	Time point	Instrument	Completed by	Delivery and time
**Outcome 1:** To compare the developmental, social-emotional and health status of children with a prenatal diagnosis of a prenatal copy number variant with those children with no copy number variant detected on chromosomal microarray.				
Quantitative survey	Children aged 12 months or older.	Brief Infant-Toddler Social and Emotional Assessment (BITSEA) [20]	Parent	Online or hard copy. 30-40 minutes total
		Strengths and Difficulties Questionnaire (SDQ) [21]	Parent	
		Maternal Postnatal Attachment Questionnaire (MPAS) [22]	Parent	
		Vulnerable Child Scale (VCS) [23]	Parent	
		Profile of Mood States (POMS) [24]	Parent	
		Parent Sense of Competence Scale (PSOC) [25]	Parent	
		Golombok Rust Inventory of Marital State (GRIMS) [26]	Parent	
		Disclosure of Results to Others [27]	Parent	
		Secrecy about Results [28]	Parent	
		Decision Satisfaction Scale [29, 30]	Parent	
		Revised Scale of Ambiguity Tolerance [31]	Parent	
		Genetic essentialism, general understanding of genetics and accuracy of understanding of results [32, 33]	Parent	
		Patient Health Questionnaire depression scale (PHQ-8)	Parent	
		Children with Special Health Care Needs (CSHCN) screener [34]	Parent	
**Outcome 2:** To measure the impact of a prenatal diagnosis of a copy number variant on maternal perceptions of their child.				
Quantitative survey	‘Younger cohort’ children who are 12-30 months old at the time of enrolment	Ages and Stages Questionnaire-3	Parent	Online or hard copy. 20-30 minutes.
		Ages and Stages Questionnaire Social Emotional-2	Parent	
Quantitative survey	‘Older cohort’ children who are 2 years 7 months to 7 years 7 months at the time of enrolment	The Vineland-II Adaptive Behavior Scale (VABS)	Parent	Online or hard copy. 20-60 minutes
Psychologist assessment		Wechsler Preschool and Primary Scale of Intelligence IV (WPPSI-IV)	Psychologist	In person. 20-60 minutes
Clinical assessment		Clinical assessment by study pediatrician: measurements of height, weight, and head circumference; history and physical examination	Paediatrician	In person. 20-60 minutes
**Outcome 3:** The frequency of copy number variants that have a change in classification after reanalysis after ≥2 years will be presented in a descriptive analysis. For the participants in the reanalysis group, an additional letter will be sent to them with the results of the updated analysis.				
Clinical standard laboratory software and databases in use during 2021-2022	Children who were prenatally diagnosed with a copy number variant of uncertain or unknown significance	Affymetrix Chromosome Analysis Suite (ChAS) software	Cytogenetics Laboratory	n/a
		DatabasE of Chromosomal Imbalance and Phenotype in Humans using Emsembl Resources (DECIPHER)	Cytogenetics Laboratory	
		International Standards for Cytogenomic Arrays (ISCA) database	Cytogenetics Laboratory	
		Online Mendelian Inheritance in Man (OMIM)	Cytogenetics Laboratory	

### Covid-19 contingency plan

Due to the COVID-19 pandemic in 2020, we developed an alternative protocol in the event we are not able to assess children face-to-face. See Supplementary Table 1 for details. At the time of submission in May 2021, the Covid-19 contingency plan was not activated.

### Follow up correspondence with participants

Follow-up correspondence with a summary report of the child’s assessments will be sent to the mother within six weeks of the completion of the Ages and Stages Questionnaires (younger cohort) or the on-site assessments (older cohort). An offer to discuss the results with the study coordinator with contact details will be provided. If the woman wishes the PALM study to share the results of the assessments with her child’s health care provider, written consent will be obtained for this information sharing.

For the participants in the CNV reanalysis group, an additional letter will be sent to them with the results of the updated analysis. Participants will be offered a clinical referral to discuss the findings if the CNV classification has changed from the original prenatal report.

### Statistical methods of analysis and power calculations

#### Objective 1:

The primary outcome for the 2–7-year-old group is the WPPSI-IV full IQ score. The WPPSI-IV is standardized to be normally distributed with a mean of 100 and standard deviation of 15 in the general population (variance of 30). It is therefore possible to perform the primary analysis as a single group comparison by t-test of the WPPSI-IV full IQ score in the children enrolled in this study with the population norm. A between-group analysis of the WPPSI-IV for the CNV study group and control (non-CNV) group cohorts will be done. The results from the children with VUS will be analyzed separately from those with pathogenic CNVs (pCNVs).

If 300 participants with VUS are enrolled, this will yield > 95% power and 95% confidence to detect a 3-point difference (0.2SD) in mean IQ from the norm assuming that the standard deviation is 15.

Our study cohort of women with VUS are a heterogenous group with varying indications for testing. As the presence of a fetal structural abnormality is likely to be a modifier of all assessed outcomes, a stratified analysis of the CNV cohort with and without this indication for prenatal diagnosis will be performed. An audit of the VPDD data shows that those with a fetal structural abnormality comprise two thirds of the VUS study cohort, leaving 100 to study without such an abnormality. The required sample size is therefore a minimum of 87 women without a fetal structural abnormality in each of the study and control cohorts to detect a 3-point difference in mean IQ (95% power, 95% confidence).

For the group with pCNVs, a larger difference in mean IQ is expected between those with pCNVs and those without any CNV. A minimum of 13 participants in the pCNVs cohort is required to detect a mean difference of 8 IQ points with 95% power and 95% confidence. It is expected that this analysis can be performed on the subgroup with the most common pCNV (22q11.2 deletion syndrome), which is detected prenatally in approximately 7 cases per year. If 100% progressed to live outcome, there would be at least 35 children in the cohort (born from 2013-2018) that would be able to perform the WPPSI-IV. Less than 50% would be required to be available for recruitment to meet the minimum sample size of 13.

#### Secondary outcomes

Vineland-II Adaptive Behavior Scale (VABS) scores, adjusted z-scores for growth, overall frequency of medical problems. As for the primary outcome, secondary outcomes will also be analyzed according to their subgroups (pCNVs and VUS). Multivariable logistic (for binary outcomes) or linear (for continuous outcomes) regression analysis will be performed to account for modifiers and confounders, such as maternal age, parental education level, gestational age at birth.

#### For the 12–24-month-old group, the ASQ-3 and ASQ:SE-2 scores are the primary outcomes

Scores for individual items are summed to give an overall continuous score for each of the domains in the ASQ-3 and ASQ:SE-2. In addition to using scores as continuous outcomes, we will categorize the scores according to the recommended scores for typical development, need for monitoring, and need for further assessment. We will collapse these three categories into a binary variable (those with typical development vs those with a need for monitoring or further assessment [i.e., potential atypical or delayed development]). The differences in those with typical and ‘atypical’ scores will be compared among the CNV subgroup using chi-squared test for proportions or Fisher’s exact test as appropriate.

#### Objective 2

The primary outcome will be the BITSEA score. We will analyze the data according to the subgroup of CNV result (pCNV, VUS) compared with controls, stratified by age. The secondary outcomes will be the Maternal Postnatal Attachment Questionnaire (MPAS), Vulnerable Child Scale (VCS), Profile of Mood States (POMS), and the Parent Sense of Competence Scale (PSOC). For the secondary outcomes, we will compare group differences in responses using mixed effect regression models. We will report mean values for the parent report of child behavior, parenting competence, attachment, perceived child vulnerability and parent mood. Parent gender, country of birth, education, employment status, and child sex will be adjusted as covariates.

#### Objective 3

We will compare the classifications given at the time of issue of the prenatal report and the current classifications in 2020-21 according to the issuing laboratory’s protocols. The proportion of pCNVs, VUS and benign variants will be compared between the prenatal and 2-year postnatal classifications using chi-squared test for proportions.

## Discussion

This cohort study addresses four current areas of uncertainty in practice, including

### Lack of choice in amount of fetal genomic information received

We have previously shown that when given a choice about receiving different levels of genomic information from prenatal CMA, 40% of Australian pregnant women choose NOT to have extended information that might include VUS [35]. However, outside this research protocol, reporting on VUS remains standard laboratory practice, irrespective of patient preference. Our study will provide new data on the postnatal outcomes of VUS which may help inform the clinical utility of revealing this information during pregnancy.

### Women’s experience of prenatal VUS

While most children with VUS are expected to have normal outcomes, the possibility of an increased risk of developmental disorder can result in parental anxiety, termination of healthy pregnancies, “hypervigilant” parenting styles, and increased utilization of medical services [36]. A prenatal diagnosis of a VUS may also alter early parental perceptions of the competency of the child [16]. Our study will be able to determine whether there are any such ‘harms’ associated with the disclosure of VUS, and enable us to develop strategies to mitigate these negative effects.

### Dealing with rapidly evolving knowledge base

Genomic knowledge is accelerating, and fetal VUS may be reclassified as benign or pathogenic as clinical evidence accumulates and annotation resources are updated. Reanalysis of CNVs in the NICHD prenatal microarray cohort after three years showed a dramatic reduction in the numbers of VUS from 94 to 35, and a corresponding increase in pathogenic CNVs (pCNVs) (from 35 to 70) and ‘likely benign’ CNVs (from 36 to 60) [37]. An Australian study of a pediatric cohort that was evaluated with CMA in 2010 showed a statistically significant increase in potentially pathogenic results from 19% to 31% upon reanalysis in 2012 [38]. There is currently no guidance on whether a prenatal CNV should be reinterpreted in the postnatal period and whose responsibility it is to contact parents in the case of a change in classification [39]. This study will provide information to help guide clinical practice recommendations about postnatal review and follow-up.

### Lack of structured follow up for children at risk of developmental disorders

There is no standardized approach to identifying and following up infants after a prenatal diagnosis of VUS in Australia. These infants may be at increased risk of poor outcome, yet there is no translation of this knowledge into clinical practice. This study will provide the outcomes required to understand the potential need for structured developmental follow-up.

### Translation plan

Translation plan for this research will depend on the final results. We anticipate that there may be new recommendations for clinical practice, including:

- Structured follow up for children diagnosed prenatally with VUS,
- Offer parents the option of re-analysis of the CMA result after 2 years postnatal age
- Development of patient information resources for pregnant women and their partners after receiving a result of uncertain significance
- Updating of existing genomic annotation resources such as DatabasE of Chromosomal Imbalance and Phenotype in Humans using Ensembl (DECIPHER) with new genotype-phenotype correlations

### Potential risks related to study conduct

#### Creation of new clinically relevant information during the study

There are several potential opportunities for information to be disclosed that may require clinical action.

#### Poorer than expected results on the developmental assessments

Participants will be given a summary report on their child’s results by email or standard mail (as preferred) with a qualitative description of their child’s scores. They will be encouraged to contact the study coordinator if they have concerns regarding the report and they may have the full results sent to their child’s healthcare provider or the education provider following receipt of a signed consent form. If the participant requires a follow-up or a new referral to a clinical service, our study pediatrician or psychologist will facilitate this for them.

Reclassification of the child’s genomic variant by the laboratory may bring benefits (e.g., reassurance if a VUS has been reclassified as benign) or potential harms (it may cause further anxiety if the presumed cause of a child’s phenotype is no longer considered contributory, or it has been reclassified from ‘uncertain significance’ to ‘likely pathogenic’). For this reason, all CNVs will be reported to the participant regardless of the ‘direction’ of reclassification (increased or decreased clinical significance, or no change in classification). The results will be sent with a cover letter with information on how to contact our study coordinator if they have any questions or concerns. The results will be shared with their nominated health care provider after signed consent to share information. If the participant requires a referral to a clinical service, our study pediatrician or psychologist will facilitate this for them.

#### Distress caused by contacting women who have experienced a child death or stillbirth

Our pre-screening steps are time-consuming and resource-intensive but essential to minimize the risk of contacting women who have had a termination of pregnancy, perinatal loss or child death. However, we cannot eliminate the possibility of accidently sending invitation letters to women whose child has died and thus potentially causing emotional distress. We have designed the most feasible prescreening process available under the current constraints.

#### Poor recruitment

We have based our uptake rates on those experienced in a similar multicenter follow up study in the United States, and therefore believe these to be realistic. However, it is possible that we will not meet our recruitment targets either due to higher-than-expected ineligibility rates, missing data, or study acceptability. If that occurs, we will endeavor to pool our data with the United States cohort as we have harmonized our outcome measures to facilitate this if needed. Permission for data sharing outside Australia has been included as an option in the consent form.

### Summary

The PALM study is the first Australian study to follow up a cohort of mother-child pairs to assess the long-term developmental, social-emotional and health outcomes of children diagnosed before birth with a genomic CNV on CMA. We expect that this will improve our care of families by providing evidence to guide pre-and postnatal medical care, and to mitigate the negative consequences of conveying genomic uncertainty during pregnancy.

## Supplementary Information

**Additional file 1: Supplementary Table 1.** Alternative study instruments in the event we are not able to assess children on site due to Covid-19.

## Data Availability

Not applicable.

## Abbreviations

AE: Adverse Event
ASQ:SE-2: Ages and Stages Questionnaire: Social-Emotional-2
ASQ-3: Ages and Stages Questionnaire, third edition
BEMAPRE: Belgium MicroArray Prenatal Microarray consortium
BITSEA: Brief Infant-Toddler Social and Emotional Assessment
BRIEF: Behavior Rating Inventory of Executive Function
BRIEF-P: Behavior Rating Inventory of Executive Function – Preschool version
ChAS: Chromosome Analysis Suite
CSHCN: Children with Special Health Care Needs screener
CMA: Chromosomal microarray
CNV: Copy number variant
CVS: Chorionic villus sampling
DECIPHER: DatabasE of Chromosomal Imbalance and Phenotype in Humans using Ensembl Resources
DGV: Database of Genomic Variation
GRIMS: Golombok Rust Inventory of Marital State
HREA: Human Research Ethics Application
HREC: Human Research Ethics Committee
ISCA: International Standards for Cytogenomic Arrays
MCRI: Murdoch Children’s Research Institute
MHW: Mercy Hospital for Women
MMC: Monash Medical Centre
MPAS: Maternal Postnatal Attachment Questionnaire
NHMRC: National Health and Medical Research Council
NICHD: National Institute of Child Health and Human Development
PALM: PrenatAL Microarray
pCNV: Pathogenic copy number variant
PHQ-8: Patient Health Questionnaire Depression scale-8
PI/CPI: Principal Investigator / Coordinating Principal Investigator
PICF: Participant Information and Consent Form
POMS: Profile of Mood States
PSOC: Parent Sense of Competence Scale
RCH: Royal Children’s Hospital (Melbourne)
RGO: Research Governance Office
RWH: Royal Women’s Hospital (Melbourne)
SDQ: Strengths and Difficulties Questionnaire
SEHQ: School Entrant Health Questionnaire
SoA: Schedule of Assessments
VABS: Vineland-II Adaptive Behavior Scale
VCGS: Victorian Clinical Genetics Services
VCS: Vulnerable Child Scale
VPDC: Victorian Perinatal Data Collection
VPDD: Victorian Prenatal Diagnosis Data collection
VUS: Variants of uncertain/unknown significance
WPPSI-IV: Wechsler Preschool and Primary Scale of Intelligence IV
