# Supplementary material for: Study protocol: childhood outcomes of fetal genomic variants: the PrenatAL Microarray (PALM) cohort study
Source: BMC Pediatr. 2021 Oct 11;21:447. doi: 10.1186/s12887-021-02809-7 (PMC8502634; doi:10.1186/s12887-021-02809-7)
Supplement: Supplementary file 1 — Additional file 1: Supplementary Table 1. Alternative study instruments in the event we are not able to assess children on site due to Covid-19. [file 12887_2021_2809_MOESM1_ESM.docx]

**Supplementary table 1:** Alternative study instruments in the event we are not able to assess children on site due to Covid-19.

| **Data collection method** | **Time point** | **Instrument** | **Completed by** | **Delivery and time** |
| --- | --- | --- | --- | --- |
| **Outcome 2:** To measure the impact of a prenatal diagnosis of a copy number variant on maternal perceptions of their child | | | | |
| Quantitative survey | Children who are 2 years 7 months to 7 years 7 months old at the time of enrolment | The Vineland-II Adaptive Behavior Scale (VABS) | Psychologist | Interview of parent via telehealth. 20-60 minutes. |
|  |  | Behavior Rating Inventory of Executive Function – Preschool version (BRIEF-P) (31 months < 5 years) | Parent | Offsite online assessment. 10-15 minutes |
|  |  | Behavior Rating Inventory of Executive Function (BRIEF) (5-7 years) | Parent | Offsite online assessment. 10-15 minutes |
| Telehealth review |  | Clinical assessment by study pediatrician | Paediatrician | Varied |
